# The Role of ACT-Like Subdomain in Bacterial Threonine Dehydratases

**DOI:** 10.1371/journal.pone.0087550

**Published:** 2014-01-24

**Authors:** Xuefei Yu, Yanyan Li, Xiaoyuan Wang

**Affiliations:** 1 State Key Laboratory of Food Science and Technology, Jiangnan University, Wuxi, China; 2 Key Laboratory of Industrial Biotechnology of Ministry of Education, School of Biotechnology, Jiangnan University, Wuxi, China; 3 Synergetic Innovation Center of Food Safety and Nutrition, Jiangnan University, Wuxi, China; La Trobe University, Australia

## Abstract

In bacteria, threonine dehydratases could convert L-threonine to 2-ketobutyrate. Some threonine dehydratases contain only a catalytic domain, while others contain an N-terminal catalytic domain and a C-terminal regulatory domain composed of one or two ACT-like subdomains. However, the role of the ACT-like subdomain in threonine dehydratases is not clear. Here, nine different bacterial threonine dehydratases were studied. Three of the nine contain no ACT-like subdomain, four of them contain a single ACT-like subdomain, and two of them contain two ACT-like subdomains. The nine genes encoding these threonine dehydratases were individually overexpressed in *E. coli* BL21(DE3), and the enzymes were purified to homogeneity. Activities of the purified enzymes were analyzed after incubation at different temperatures and different pHs. The results showed that threonine dehydratases with a single ACT-like subdomain are more stable at higher temperatures and a broad range of pH than those without ACT-like subdomain or with two ACT-like subdomains. Furthermore, the specific activity of threonine dehydratases increases with the increase of the number of ACT-like subdomains they contain. The results suggest that the ACT-like subdomain plays an important role in bacterial threonine dehydratases.

## Introduction

**Figure 1:**
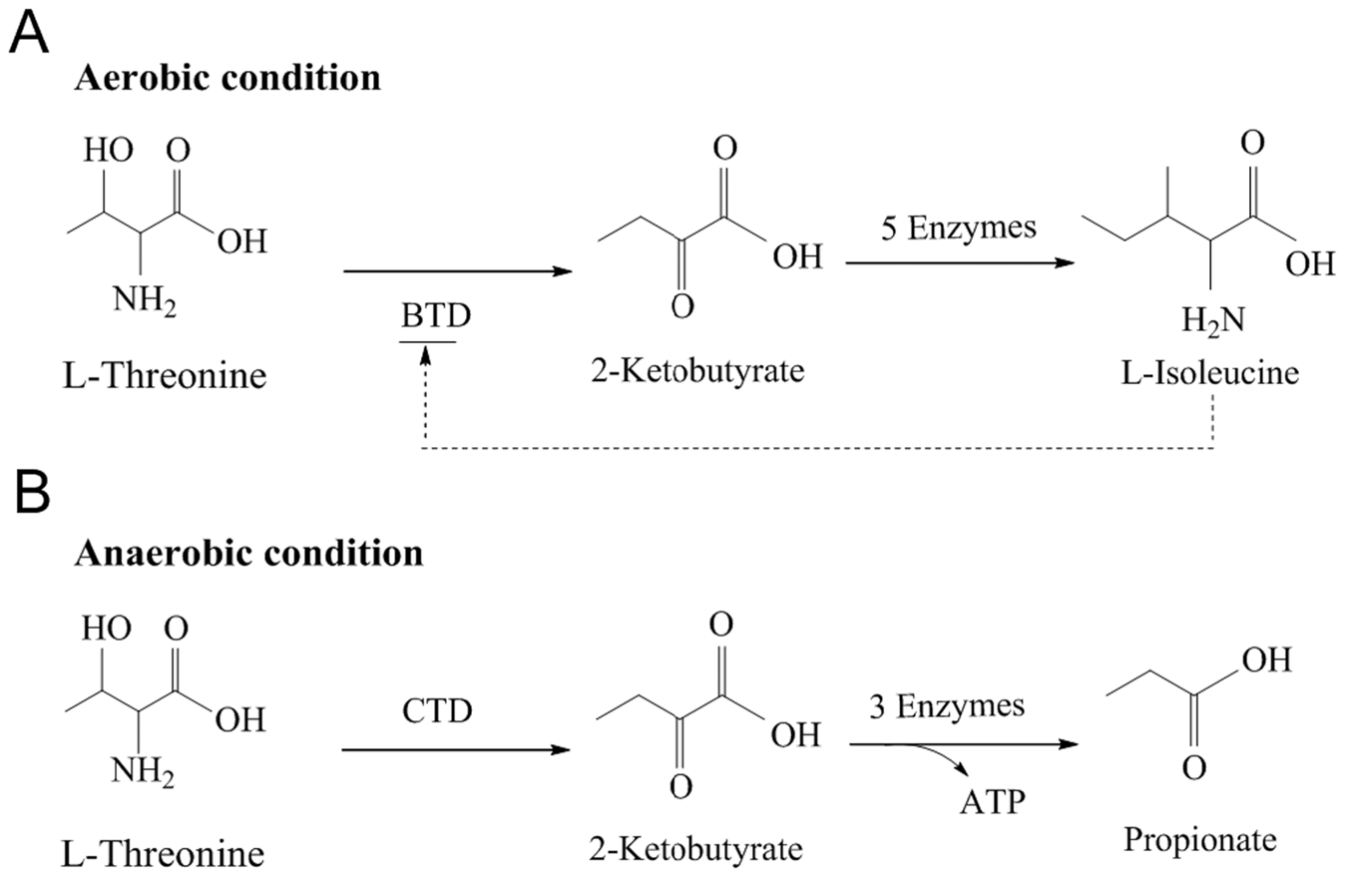
The two different pathways BTD and CTD involved in bacteria. A. BTD catalyzes the first reaction in the biosynthesis of L-isoleucine in bacteria under aerobic conditions. BTD is feedback inhibited by L-isioleucine. B. CTD degrades L-threonine to propionate in bacteria under anaerobic conditions to generate ATP. CTD is activated by AMP and CMP.

Bacterial threonine dehydratase (TD) converts L-threonine to 2-ketobutyrate. There are usually two types of TD: biosynthetic threonine dehydratase (BTD) and catabolic threonine dehydratase (CTD) (Fig. 1). BTD functions in the biosynthetic pathway of L-isoleucine when bacteria grow under aerobic conditions, and usually contains an N-terminal catalytic domain and a C-terminal regulatory domain. CTD contains only the catalytic domain, and plays a role in the degradation of L-threonine to propionate when bacteria grow under anaerobic conditions [1], its activity is activated by AMP and CMP [2].

ACT (named after the first letters of three proteins, Aspartate kinase, Chorismate mutase and TyrA) is a structural motif in proteins that function in the control of metabolism, solute transport, and signal transduction [3]. BTD encoded by the gene *ilvA* in *Escherichia coli* contains two ACT-like subdomains in its C-terminal regulatory domain. The activity of the enzyme is inhibited by the end product L-isoleucine but activated by L-valine, the product of a competing biosynthetic pathway [4]. BTD encoded by *ilvA* in *Bacillus subtilis*, however, contains only one ACT-like subdomain in its C-terminal regulatory domain, and is inhibited by L-isoleucine or by high concentrations of L-valine [5]. The interaction between different domains may facilitate enzyme foldingand increase stability [6]. Since BTD is a key enzyme for improving L-isoleucine production in bacterial fermentation [7]-[9], it is necessary to investigate the role of the ACT-like subdomain in the enzyme.

In this study, nine TDs containing different numbers of ACT-like subdomains were chosen from six different bacterial strains. The nine genes encoding these TDs were individually overexpressed in *E. coli*, and activities of the purified enzymes were analyzed after they were incubated at different temperatures and different pHs. The results indicate that the ACT-like subdomain plays an important role in the activity of TDs in bacteria.

## Materials and Methods

### Bacterial strains, plasmids and DNA manipulation

All bacterial strains and plasmids used in this study are listed in Table 1. *E. coli* JM109 was used for constructing plasmids, while *E. coli* BL21 (DE3) was used for expressing TD enzymes. Plasmid pET28a was selected as the vector for protein expression. All *E. coli* cells were grown at 37°C in LB media (1% peptone, 0.5% yeast extract, 0.5% NaCl).

**Table 1:** Bacterial strains and plasmids used in this work.

Strain or plasmid	Description	Sources
***Strains***		
JM109	Wide type *E. coli*	Stratagene
BL21(DE3)	*E. coli* for protein expression	Stratagene
W3110	*E. coli* W3110	Stratagene
ATCC 13032	*C. glutamicum*	ATCC
Mu50	*Staphylococcus aureus*	Stratagene
NBRC 13350	*Streptomyces griseus*	Stratagene
str. 168	*Bacillus subtilis*	Stratagene
KT2440	*Pseudomonas putida*	Stratagene
BL21(DE3)/pET28a-*CgtdcB*	BL21(DE3) harboring pET28a-*CgtdcB*	This work
BL21(DE3)/pET28a-*EctdcB*	BL21(DE3) harboring pET28a-*EctdcB*	This work
BL21(DE3)/pET28a-*SatdcB*	BL21(DE3) harboring pET28a-*SatdcB*	This work
BL21(DE3)/pET28a-*CgilvA*	BL21(DE3) harboring pET28a-*CgilvA*	This work
BL21(DE3)/pET28a-*SailvA*	BL21(DE3) harboring pET28a-*SailvA*	This work
BL21(DE3)/pET28a-*SgilvA*	BL21(DE3) harboring pET28a-*SgilvA*	This work
BL21(DE3)/pET28a-*BsilvA*	BL21(DE3) harboring pET28a-*BsilvA*	This work
BL21(DE3)/pET28a-*EcilvA*	BL21(DE3) harboring pET28a-*EcilvA*	This work
BL21(DE3)/pET28a-*PpilvA*	BL21(DE3) harboring pET28a-*PpilvA*	This work
***Plasmids***		
pET28a	Expression vector in *E. coli*	
pET28a-*CgtdcB*	pET28a harboring *C. glutamicum tdcB*	This work
pET28a-*EctdcB*	pET28a harboring *E. coli tdcB*	This work
pET28a-*SatdcB*	pET28a harboring *S. aureus tdcB*	This work
pET28a-*CgilvA*	pET28a harboring *C. glutamicum ilvA*	This work
pET28a-*SailvA*	pET28a harboring *S. aureus ilvA*	This work
pET28a-*SgilvA*	pET28a harboring *S. griseus ilvA*	This work
pET28a-*BsilvA*	pET28a harboring *B. subtilis ilvA*	This work
pET28a-*EcilvA*	pET28a harboring *E. coli ilvA*	This work
pET28a-*PpilvA*	pET28a harboring *P. putida ilvA*	This work

All the primers used for the PCR amplifications are listed Table 2. *CgtdcB* was amplified from the genomic DNA of *C. glutamicum*, using the primer pair of CgCTD-F and CgCTD-R; *EctdcB* was amplified from the genomic DNA of *E. coli*, using the primer pair of EcCTD-F and EcCTD-R; *SatdcB* was amplified from the genomic DNA of *S. aureus*, using the primer pair of SaCTD-F and SaCTD-R; *CgilvA* was amplified from the genomic DNA of *C. glutamicum*, using the primer pair of CgBTD1-F and CgBTD1-R; *SailvA* was amplified from the genomic DNA of *S. aureus*, using the primer pair of SaBTD1-F and SaBTD1-R; *SgilvA* was amplified from the genomic DNA of *S. griseus*, using the primer pair of SgBTD1-F and SgBTD1-R; *BsilvA* was amplified from the genomic DNA of *B. subtilis*, using the primer pair of BsBTD1-F and BsBTD1-R; *EcilvA* was amplified from the genomic DNA of *E. coli*, using the primer pair of EcBTD2-F and EcBTD2-R; *PpilvA* was amplified from the genomic DNA of *P. putida*, using the primer pair of PpBTD2-F and PpBTD2-R. All PCR amplifications were carried out in a 50 µL reaction mixture containing 5 µL 10× Extaq buffer, 4 µL dNTP mixture (2.5 mM each), 1 µL template DNA, 1 µL forward primer (20 µM), 1 µL reverse primer (20 µM) and 0.5 µL Extaq. The PCR product was digested by restriction enzymes, ligated into pET28a which was similarly digested, transformed into *E. coli* JM109 and selected on LB plates containing 50 µg/mL kanamycin. The correct recombinant plasmids were then purified and transformed into *E. coli* BL21(DE3), resulting in nine *E. coli* strains: BL21(DE3)/pET28a-*CgtdcB*, BL21(DE3)/pET28a-*EctdcB*, BL21(DE3)/pET28a-*SatdcB*, BL21(DE3)/pET28a-*CgilvA*, BL21(DE3)/pET28a-*SailvA*, BL21(DE3)/pET28a-*SgilvA*, BL21(DE3)/pET28a-*BsilvA*, BL21(DE3)/pET28a-*EcilvA* and BL21(DE3)/pET28a-*PpilvA*.

**Table 2:** The primers used for the PCR amplifications in this work.

Primers	Sequences(5′-3′)	Restriction site
CgCTD-F	CCGGAATTCATGCTCACCCTCAACGATGTC	EcoRI
CgCTD-R	CCCAAGCTTTCACAGTGTTGTGAGGTCAGT	HindIII
EcCTD-F	CCGGAATTCATGCATATTACATACGATCTGCC	EcoRI
EcCTD-R	CCCAAGCTTTTAAGCGTCAACGAAACCGGT	HindIII
SaCTD-F	CCGAATTCATGACAACCAACACAGTTACAT	EcoRI
SaCTD-R	CCGCTCGAGTTAACCTACCACACCCTTACTT	XhoI
CgBTD1-F	GGAATTCCATATGAGTGAAACATACGTGTCTGAG	NdeI
CgBTD1-R	CGGGATCCTTAGGTCAAGTATTCGTACTCAG	BamHI
SaBTD1-F	CCGAATTCATGACAGTCAAAACAACAGTT	EcoRI
SaBTD1-R	CCGCTCGAGTTAGATTAACAATGAATATAACATCTT	XhoI
SgBTD1-F	CATGCCATGGTGAGCTTCCGCGCGACCG	NcoI
SgBTD1-R	CCGCTCGAGGCCCATCACCCGGAAGCC	XhoI
BsBTD1-F	CCGAATTCATGAAACCGTTGCTTAAAGAA	EcoRI
BsBTD1-R	CCGCTCGAGTTAGATTAGCAAATGGAACAAG	XhoI
EcBTD2-F	CCCGGATCCATGGCTGACTCGCAACCCCT	BamHI
EcBTD2-R	CCGCTCGAGCTAACCCGCCAAAAAGAACCT	XhoI
PpBTD2-F	CGGGATCCATGCTCGAACAGTACGTCAAG	BamHI
PpBTD2-R	CCACTCGAGTCAGCCCAGGAACAGTTTGT	XhoI

The recognition sites for restriction enzymes are underlined.

### Expression and purification of nine different TD enzymes

TD enzymes CgCTD, EcCTD, SaCTD, CgBTD1, SaBTD1, SgBTD1, BsBTD1, EcBTD2 and PpBTD2 were purified from *E. coli* BL21(DE3)/pET28a-*CgtdcB*, BL21(DE3)/pET28a-*EctdcB*, BL21(DE3)/pET28a-*SatdcB*, BL21(DE3)/pET28a-*CgilvA*, BL21(DE3)/pET28a-*SailvA*, BL21(DE3)/pET28a-*SgilvA*, BL21(DE3)/pET28a-*BsilvA*, BL21(DE3)/pET28a-*EcilvA* and BL21(DE3)/pET28a-*PpilvA*, respectively. Each strain was grown in LB media at 25°C. Isopropyl-β-D-thiogalactopyranoside was added to a final concentration of 0.5 mM when OD_600_ of the cells reached 0.6. The cells were further grown for 10 h and harvested by centrifugation at 3750×g for 20 min. The cell pellets were resuspended in the binding buffer (20 mM HEPES, 500 mM NaCl, 20 mM imidazole, pH 8.0), and disrupted by an ultrasonic disintegrator in an icy bath for 15 min. Cell debris was removed by centrifugation at 13000×g for 20 min, and the crude extracts were used for TD purification with a Ni-NTA Sepharose column (QIAGEN). The column was firstly washed with 10 volumes of the binding buffer, applied the crude extracts, washed again with 10 volumes of the binding buffer, and then the TD enzyme was eluted with 5 volumes of the elution buffer (20 mM HEPES, 500 mM NaCl, 500 mM imidazole, pH 8.0). The purified TD enzymes were stored at 4°C in the elution buffer. Protein concentration was determined using the Bradford method [10]. The expression level and purity of the enzymes were analyzed by using 12% SDS-PAGE.

### Activity assays of TD enzymes at different temperatures and pH

Enzyme activities of the purified TD enzymes were measured according to the published method [11]. The 1 mL reaction mixture contains 100 mM potassium phosphate buffer, pH 8.0, 40 mM threonine, 1 mM pyridoxal phosphate and 2 µg enzymes. The reaction was started by adding L-threonine, incubated at 22°C for 15 min, and then terminated by adding 1 mL stopping solution (1% semicarbazide and 0.9% sodium acetate). The amount of 2-ketobutyrate in the reaction mixture was determined by measuring the absorption at 254 nm (*ε* = 0.52 mmol/cm) using a UV/Vis spectrophotometer. One unit activity was defined as the enzyme amount required for the formation of 1 mole 2-ketobutyrate per minute at 22°C, and the specific activity was defined as the total activity per mg protein.

To measure the activity at different temperatures, the purified enzymes were incubated at the specific temperature for 10 min, and then cooled on ice. The *T_50_* values were measured as described in the published method [11].

To analyze the enzyme activity at different pH, the purified enzyme was first diluted 10 times in acetic acid/sodium acetate buffer (pH 5), phosphate buffer (pH 6–8), or glycine sodium hydroxide buffer (pH 9–11), and incubated on ice for 30 min, before assaying the enzymes activity in 100 mM potassium phosphate buffer (pH 8.0).

## Results and Discussion

**Figure 2:**
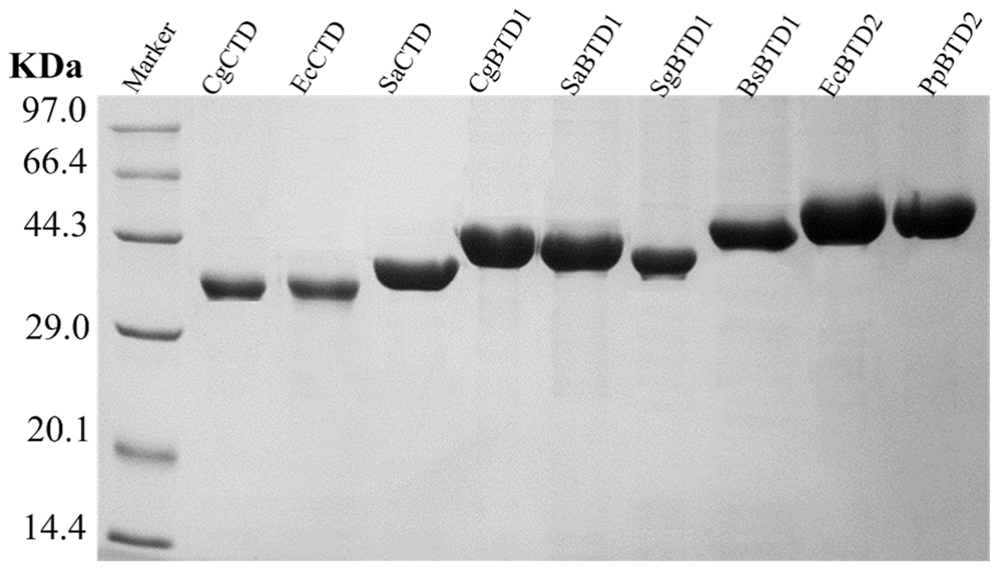
SDS-PAGE of the nine purified TDs. Lane M, molecular mass marker; lane 1, CgCTD purified from BL21(DE3)/pET28a-*CgtdcB*; lane 2, EcCTD purified from BL21(DE3)/pET28a-*EctdcB*; lane 3, SaCTD purified from BL21(DE3)/pET28a-*SatdcB*; lane 4, CgBTD1 purified from BL21(DE3)/pET28a-*CgilvA*; lane 5, SaBTD1 purified from BL21(DE3)/pET28a-*SailvA*; lane 6, SgBTD1 purified from BL21(DE3)/pET28a-*SgilvA*; lane 7, BsBTD1 purified from BL21(DE3)/pET28a-*BsilvA*; lane 8, EcBTD2 purified from BL21(DE3)/pET28a-*EcilvA*; lane 9, PpBTD2 purified from BL21(DE3)/pET28a-*PpilvA*.

In order to investigate the role of ACT-like subdomains in TD enzymes of bacteria, nine genes encoding TD enzymes in different bacteria were cloned in pET28a and overexpressed in *E. coli*. According to the analysis of CDART (Conserved Domain Architecture Retrieval Tool) [12], CgCTD, EcCTD and SaCTD contain no ACT-like subdomain; CgBTD1, SaBTD1, SgBTD1 and BsBTD1 all contain a single ACT-like subdomain; while EcBTD2 and PpBTD2 both contain two ACT-like subdomains (Table 3). The size of the protein increases with the increase of the number of ACT-like subdomains it contains. Nine TD enzymes were overexpressed in *E. coli* and purified to homogeneity. The purity of the proteins was confirmed by SDS-PAGE analysis (Fig. 2). The migrating rates of the nine enzymes in the gel are consistent well with their theoretical size (Table 1).

**Table 3:** Information of TDs from different bacteria.

Enzyme	GI	Strain source	ACT numbers	MW (KDa)
CgCTD	gi|19552203	*C. glutamicum*	0	32.0
EcCTD	gi|388479115	*E. coli*	0	35.2
SaCTD	gi|15924428	*S. aureus*	0	37.1
CgBTD1	gi| 23308897	*C. glutamicum*	1	46.5
SaBTD1	gi|282917408	*S. aureus*	1	46.9
SgBTD1	gi|182436361	*S. griseus*	1	42.6
BsBTD1	gi|16079236	*B. subtilis*	1	46.6
EcBTD2	gi|388479474	*E. coli*	2	56.1
PpBTD2	gi|26991825	*P. putida*	2	54.9

**Figure 3:**
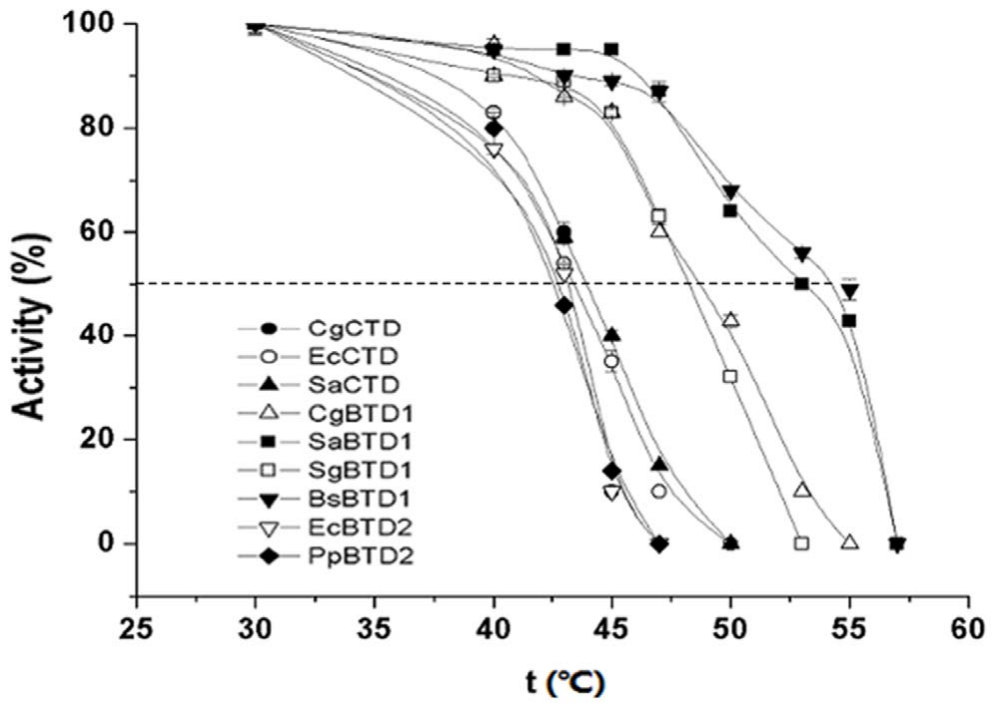
The effect of temperature on the activity of nine different TDs. Error bars indicate the standard deviations from three parallel samples.

The activity of each TD enzymes was analyzed after the enzyme was incubated for 10 min at 30, 40, 43, 45, 47, 50, 53, 55, 57 and 60°C, respectively (Fig. 3). The activities of EcBTD2 and PpBTD2 significantly decreased with the increase of temperature, and their activities were totally lost when incubating at 47°C for 10 min. The activity loss of CgCTD, EcCTD and SaCTD showed the similar level to that of EcBTD2 and PpBTD2; the activities of CgBTD1, SaBTD1, SgBTD1 and BsBTD1, however, decreased much slower than that of EcBTD2 and PpBTD2 with the increase of temperature. The *T_50_* value was around 43°C for CgCTD, EcCTD, SaCTD, EcBTD2 and PpBTD2, but ranged between 47-55°C for CgBTD1, SaBTD1, SgBTD1 and BsBTD1 (Table 4). These data reveal that at higher temperatures BTD1 is usually more stable than CTD and BTD2, suggesting that the TD enzyme containing a single ACT-like subdomain is more stable at higher temperatures.

**Table 4:** Stabillity of TDs from different bacteria.

Enzyme	CgCTD	EcCTD	SaCTD	CgBTD1	SaBTD1	SgBTD1	BsBTD1	EcBTD2	PpBTD2
T_50_ (°C)	43	43	43	47	53	47	55	43	43
Specific Activity (U/mg)	8.5	9.9	1.47	89	7.4	1.0	23	171	106

The specific activity at 30°C for each TD enzyme was also determined (Table 4). The highest specific activity for CTD, BTD1 and BTD2 was 9.9, 89 and 171 U/mg, respectively, suggesting that ACT-like subdomains could increase the specific activity of TD enzyme. Interestingly, though the BTD2 has one more ACT-like subdomain, its stability at higher temperatures is lower than that of BTD1 and equal to that of CTDs. Except for the higher specific activity, structural flexibility might be another benefit of TDs containing multiple ACT-like subdomains such as BTD2. The mechanical flexibility is critical for the function of proteins [13], and may be a promoter for generating BTD2 to adapt bacteria for the more complex environment [14], [15].

**Figure 4:**
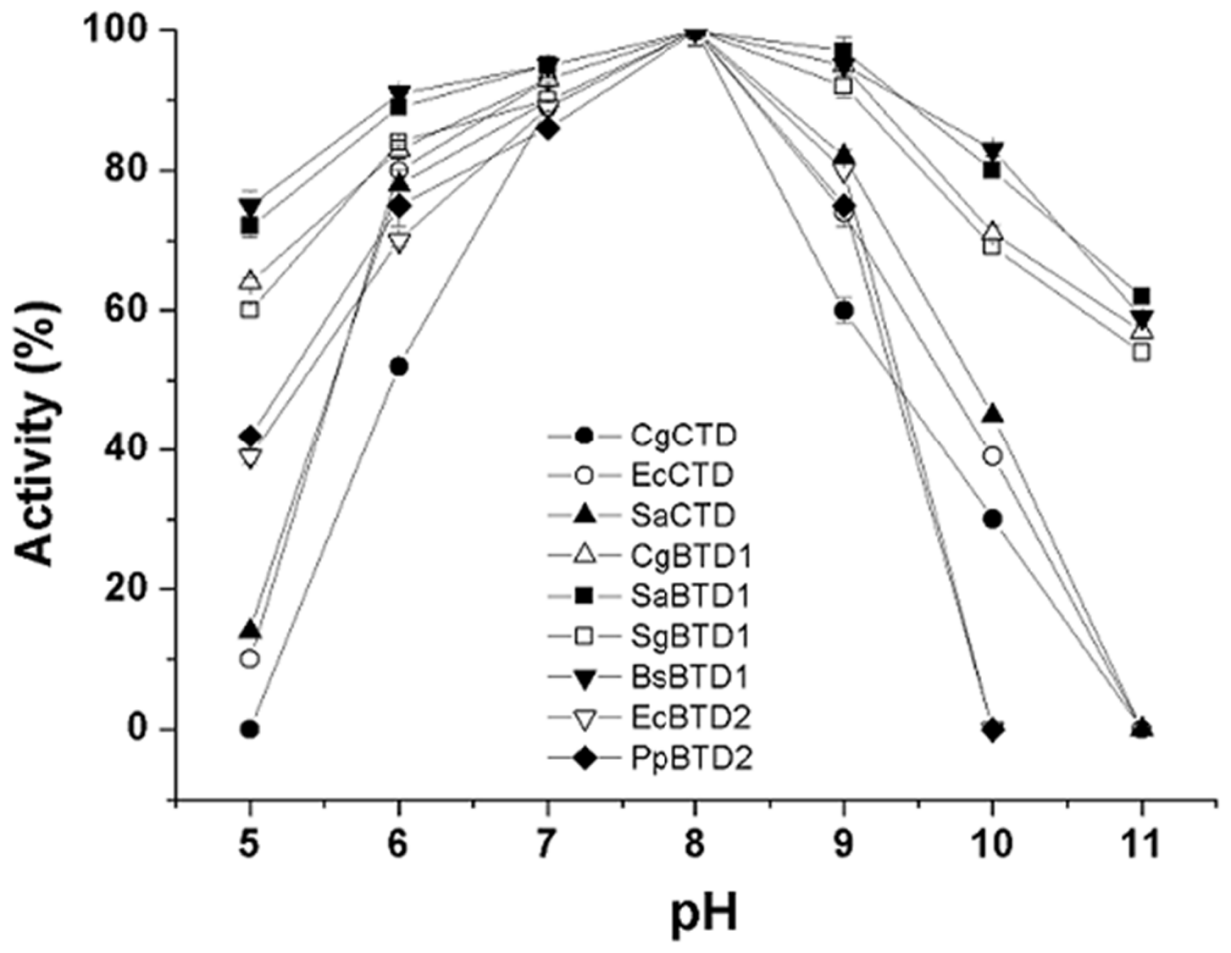
The effect of pH on the activity of nine different TDs. The remaining activities of nine TDs were measured at pH

The activities of the nine TD enzymes were also analyzed after incubation for 30 min in buffers with different pH. For all nine TDs, the remaining activity after incubating at pH 8.0 was maximal and that incubation at various other pH values prior to assaying at pH 8.0 affected the activities of the TDs in the manner shown in Fig. 4. After incubation at pH ranges from 5 to 11, the remaining activities of CgBTD1, SaBTD1, SgBTD1 and BsBTD1 were more than 60%, and were higher than those of CgCTD, EcCTD, SaCTD, EcBTD2 and PpBTD2. After incubation at pH range from 6 to 9, all the nine TD enzymes maintained more than 50% of their activities. These data reveal that BTD1 with a single ACT-like subdomain could function better at a broader range of pH than CTD and BTD2.

ACT family domain is widely conserved in bacteria and evolutionarily mobile. It is always combined with other domains to provide easily regulated enzymes [3], [16]. Most ACT domain-containing proteins have been discovered in Bacteria and Archaea, indicating that the domain appeared a long time ago [6], [17], [18]. CTD exists not only in bacteria, but also in plants and yeast [19], [20], suggesting that the pathway of L-threonine degradation may exist in the ancestral cell before the divergence of the three kingdoms. In the primordial cell where organic compounds were rich, catabolic pathways may have been more prevalent than biosynthetic pathways, therefore, only CTD may have been required for gaining energy under anaerobic conditions [17]. With the increase of the number of primordial cells, the prebiotic supply of amino acids may have been exhausted, and thus 2-ketobutyrate produced by CTD was possibly used for L-isoleucine biosynthesis. Therefore, BTD were created by combining CTD and ACT-like subdomain to better adapt the environment [21]. The hypothesis that multiple ACT domains promote TD flexibility requires further study to confirm.
